# Clinical Manifestations of Temporomandibular Disorders and Their Relationship with Sleep Disturbances and Emotional Disorders in a Spanish Pediatric Population: A Cross-Sectional Study

**DOI:** 10.3390/jcm14103599

**Published:** 2025-05-21

**Authors:** Fanny Esther Tapia-Sierra, Jesús Miguel Ticona-Flores, Guillermo Reichard-Monefeldt, Naomi Elvira-Tapia, Nuria Esther Gallardo-López, Montserrat Diéguez-Pérez

**Affiliations:** 1 Dental Clinical Specialties Department, Faculty of Dentistry, Universidad Complutense de Madrid, 28040 Madrid, Spain; ftapia01@ucm.es (F.E.T.-S.); jticona@ucm.es (J.M.T.-F.); greichar@ucm.es (G.R.-M.); negallar@ucm.es (N.E.G.-L.); 2 Preclinical Dentistry Department, Faculty of Biomedicine and Health Sciences, Universidad Europea de Madrid, 50009 Zaragoza, Spain; 3 Faculty of Health and Sports Sciences, Universidad de Zaragoza, 28670 Madrid, Spain; montserd@ucm.es

**Keywords:** temporomandibular disorders, adjustment sleep disorders, emotional aspects, pediatric dentistry

## Abstract

The etiology of temporomandibular disorders (TMDs) has been linked to various factors, including functional and psychological factors, which makes it difficult to identify associations between a single etiological factor and the signs and symptoms of TMDs. **Objectives**: This study aimed to describe the presence of TMD symptoms and their relationship with sleep disturbances and emotional disorders in children and adolescents. **Methods**: This observational study included Spanish children aged between 8 and 13 years. The measurement instruments consisted of the BRUNI survey for sleep disorders and the Educational-Clinical Questionnaire: Anxiety and Depression (CECAD) survey. A clinical examination was subsequently performed following the DC/TMD guidelines for diagnosing TMDs. Frequencies, means, and standard deviations were applied, along with the prevalence ratio as a measure of association and the chi-square test. The significance level was set at 5%. **Results:** A total of 128 participants participated in the study with a mean age of 10.89 (±2.15) years. The prevalence of TMDs was 54%, while the most common symptoms were muscle pain at 26%, joint pain at 14%, and a combination of both at 14%. Children who presented muscle pain had a mean anxiety score of 44.87 (±11.85), whereas those without symptoms had a mean score of 36 (±10.78, and 0.03 *p*-value). The BRUNI index revealed that 78.13% of patients with TMDs had difficulty initiating and maintaining sleep, with a prevalence ratio (PR) of 3.57 (*p*-value 0.041). **Conclusions**: The present study reveals that temporomandibular disorders are common in children and adolescents, with 54% presenting at least one clinical sign or symptom. Emotional disturbances and sleep problems were also prevalent, affecting 41% and 56% of participants, respectively. Early interdisciplinary screening is essential to manage the co-occurrence of TMDs, emotional distress, and sleep problems in children.

## 1. Introduction

The term temporomandibular disorders refers to disorders characterized by pain in the preauricular area and/or masticatory muscles, the presence of sounds in the TMJ during mastication, and deviations or restrictions of mandibular movements [1]. TMDs comprise a diverse range of terminology that encompasses a group of clinical problems related to the muscles of mastication, the temporomandibular joint, and associated structures. They represent the most common cause of non-dental orofacial pain and are due to multifactorial etiologies that are still under investigation [2,3]. TMDs have been linked to various factors, including functional and psychological factors, which makes it difficult to identify the association between a single etiological factor and the signs and symptoms of TMDs [4].

There are numerous epidemiological studies in the scientific literature regarding TMDs which report that they represent a condition that frequently affects adults; however, in the child and adolescent population, existing studies are insufficient. Moreover, research reflects an increase in the prevalence of this disorder with increasing age [5,6,7].

When addressing temporomandibular disorders (TMDs) in the developmental age, it is essential to employ internationally recognized diagnostic criteria that consider both somatic and psychosocial dimensions of the disorders. The most widely accepted diagnostic framework is the Diagnostic Criteria for Temporomandibular Disorders (DC/TMD), developed by the International Network for Orofacial Pain and Related Disorders Methodology. The DC/TMD employs a dual-axis approach: Axis I includes physical diagnoses such as myalgia, arthralgia, and disc displacement, while Axis II assesses psychological factors and pain-related disability [8,9].

Parafunctional habits are defined as repetitive, involuntary behaviors involving the masticatory system that are not related to normal functions like chewing, speaking, or swallowing. Common examples include awake bruxism (clenching or grinding teeth while awake), sleep bruxism (involuntary grinding during sleep), nail biting, lip or cheek chewing, and pencil biting [10,11].

These behaviors are particularly frequent during childhood and adolescence, and are considered contributing factors to the development and persistence of TMD symptoms [12,13].

Sleep disorders, especially sleep bruxism, are masticatory muscle activities that occur during sleep and are classified as sleep-related movement disorders [14]. Sleep bruxism in children has been associated with arousals, stress, and psychosocial factors, and may co-occur with TMDs in certain cases. These disturbances can exacerbate jaw pain, fatigue, and joint noises, and are important to consider in both diagnosis and treatment planning for TMDs [12,13].

Signs and symptoms of TMDs can be observed with a wide frequency range. Valesan et al. report a prevalence in young adults of 31% and in children and adolescents of 11% [5]. De Sena et al. described a general prevalence of TMDs in children ranging from 16% up to 68% [1]. Without being able to reach a consensus, the interpretation of signs and symptoms of TMDs in children comes from the understanding of these disorders as variations within the normal pattern, as preclinical characteristics, or as manifestations of a disease [5].

In addition, TMD and bruxism share a multifactorial etiology [15,16]. According to a recent meta-analysis, the estimated worldwide co-occurrence of bruxism and TMDs is 17%, with significant differences observed between geographical continents, being as high as 70% in North America and as low as 9% in Asia. In Europe this co-occurrence is 14%. A previous study demonstrated that sex was a significant factor, as the risk of co-occurrence of TMDs and bruxism was higher in females. In addition, the analysis revealed that the mean prevalence of TMDs among subjects with bruxism was 63.5%, with the highest frequency found in North America (98.3%) and the lowest in Asia (53.9%). However, the meta-analysis revealed that temporal factors and the average age of subjects did not significantly contribute to observed variability across investigations [17].

The variability in these reports may be attributed to the diversity of diagnostic criteria, different examination protocols, and the diversity of the sample [15,16]. Valesan et al. concluded that the frequency of TMDs was 11.3% in children and adolescents. In addition, the most frequent clinical manifestation was the displacement of the articular disc (8.3%), followed by the degeneration of the joint (0.4%). The displacement of the disc with reduction was the most prevalent (25.9%) TMD in young adults, with a 7.4% prevalence in children and adolescents [5]. According to a meta-analysis conducted by Zielinski et al., the prevalence of TMDs worldwide is high and affects around 34% of the population. However, the prevalence of TMDs in children and adolescents is 27% and differs depending on geographic location, varying by continent: 37% in North America, 33% in South America, 18% in Europe, and 32% in Asia [17]. It occurs in a wide age range, although, according to recent research, it most commonly develops in subjects between 20 and 40 years old [18].

In a study conducted with Brazilian adolescents, Aravena et al. reported that pain located in areas close to the temporomandibular joint was the most frequent clinical manifestation of TMDs (41%), followed by the sensation of dental clenching (32%) and joint noise (25%) [7]. Other studies in non-Caucasian populations such as Marpaug et al. showed a higher percentage of signs such as joint noises (15%) [12]. However, according to Aravena et al., the symptomatology associated with TMDs was lower (30%) [7]. However, De Sena et al. reported that in developing populations, the presence of signs can reach 68%, while only 41% experience symptoms [1].

Marpaug et al. reported that the prevalence rates of TMDs related to joint and muscle pain in children and adolescents were 23.4% and 36.9%, respectively. They also described psychological factors and sleep disorders as potential risk factors for TMDs [12]. Corroborating this, Yap et al. stated that psychological factors and the presence of bodily pain in developing patients are strongly associated with TMDs, as are oral habits that are most common in children, such as sleep bruxism, and wake bruxism in adolescents [11,13].

Al-Khotani et al. reported that 27.2% of children are diagnosed with at least one TMD symptom, with myofascial pain being the most common diagnosis (15%), followed by disc displacement with reduction, arthralgia, myofascial pain with limitation of mouth opening, and osteoarthritis. Children diagnosed with myofascial pain more frequently report orofacial pain, headaches, and teeth clenching, whereas those with arthralgia more frequently report orofacial pain and grinding compared to those without a TMD diagnosis. Self-reported orofacial pain and headache, as well as bruxism, are therefore associated with a diagnosis of disc pain and displacement [19]. In children and adolescents, TMD symptoms have been associated with certain orofacial conditions, particularly neck pain. In contrast, TMD symptoms have only been weakly associated with anxiety. Postural imbalance is not significantly related to TMD symptoms, although habitual daytime clenching significantly affects the manifestation of TMD symptoms [20].

However, despite the copious scientific literature in this area, there is no conclusive data in this regard for the Spanish child and adolescent population. The associations between TMDs and sleep disorders and psychological factors such as stress and anxiety are still not addressed. Based on the above, the objective of this cross-sectional study was to describe the frequency of clinical manifestations linked to temporomandibular disorders and their association with sleep disturbances and emotional disorders. The null hypothesis is therefore that clinical manifestations cannot be linked to temporomandibular disorders and there is no association between TMDs and sleep disturbances and emotional disorders.

## 2. Materials and Methods

### 2.1. Design and Ethical Aspects

A multicenter, observational, cross-sectional, pilot study was designed and conducted in the provinces of Guadalajara and Toledo, both in the Autonomous Community of Castilla-La Mancha. It was approved by the Ethics Committee of the Hospital Clínico San Carlos in Madrid (internal code: 21/376-E) and complied with Spanish Organic Law 3/2018 on Personal Data Protection and the Guarantee of Digital Rights.

Participants were informed about the purpose of the study, and their parents authorized their participation by signing a consent form. All data were encrypted and hidden from anyone outside the research group. The principal investigator was responsible for retaining the data and ensuring data security.

### 2.2. Study Population

The study population consisted of children between 8 and 13 years of age who attended routine dental check-ups and growth and development control appointments at public health centers and private dental clinics in the areas of Azuqueca de Henares, Alovera, and Seseña. These patients had no recent history of dental caries treatment and were invited to participate in the study as part of their regular dental visits.

#### 2.2.1. Eligibility Criteria

Children and adolescents who agreed to participate in the study and whose reading ability allowed them to complete questionnaires were included. Those with a mental disability, a degenerative systemic disease that could affect the muscular or joint system, or a history of congenital developmental disorders involving the maxillofacial complex were excluded. Likewise, patients with uncooperative behavior or a history of orthodontic or orthopedic treatment were not included in the study.

#### 2.2.2. Sample Size

The sample size was calculated using the formula for a finite population. To determine the size of the potential participating population, the corresponding number was extracted from the Spanish National Institute of Statistics website (children aged 10 years), considering the mean age of the target population. A prevalence of 21.83% reported in the study by Macrì et al. [21] was used. In addition, a significance level of 0.05, a type II error (β) of 80%, and a power of 20% were established. With these parameters, the sample calculation resulted in a sample size of 157 participants.

#### 2.2.3. Data Collection System

Informed consent was obtained from the parents and verbal assent from the patient at the research center. Each child’s guardians subsequently completed the questionnaires in the waiting room. The patient was then invited to a clinic alone to complete the emotional disturbance questionnaire so that their responses would not be influenced by the presence of a parent or legal guardian.

**Figure 1:**
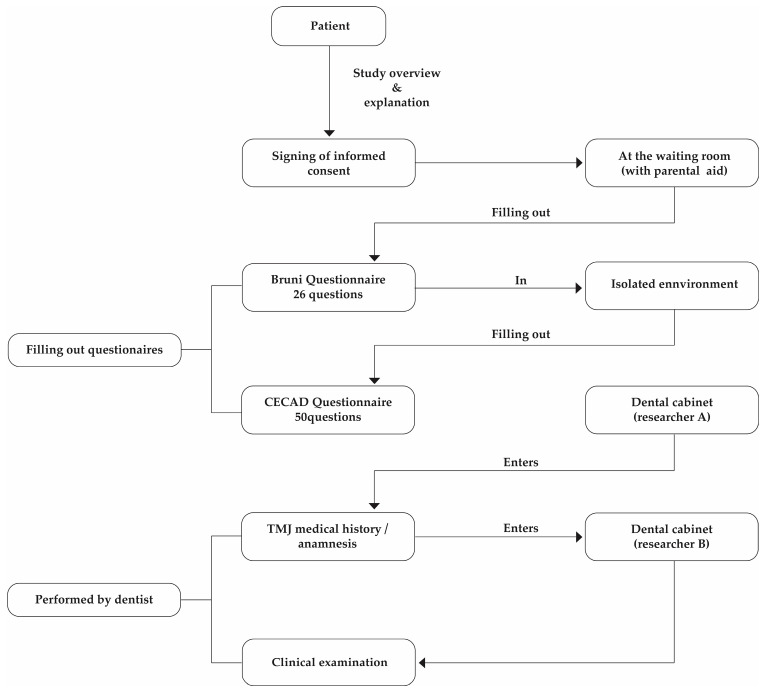
Data collection process.

In the next phase, an experienced dentist conducted a clinical examination to identify signs and symptoms associated with TMDs. This process is diagramed and presented in Figure 1.

##### Questionnaires

Information on sleep and emotional disorders was collected using two validated instruments: the Sleep Disturbance Scale for Children (SDSC), also known as the BRUNI index [22], and the Educational-Clinical Questionnaire: Anxiety and Depression (CECAD) [23].

**Figure 2:**
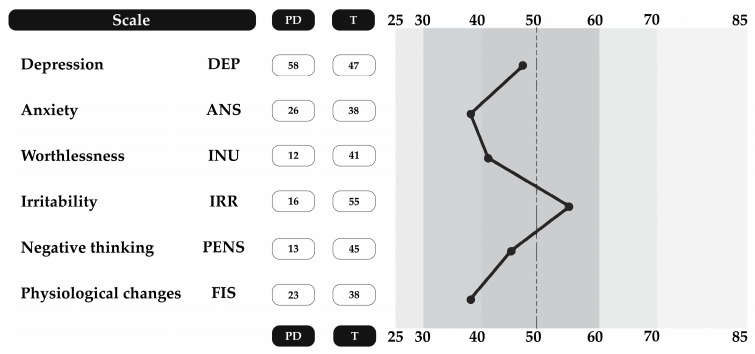
Results processed using CECAD survey virtual platform.

- Sleep Disorders:
  The SDSC/BRUNI index is a validated questionnaire designed to assess sleep disturbances in children [22]. This questionnaire consists of 26 questions that assess the following parameters: initiating and maintaining sleep, sleep breathing disorders, arousal/nightmare, sleep/wake transitions, excessive somnolence, and sleep hyperhidrosis. All questions are answered along with the parent or guardian and scored on a 5-point Likert scale (1 = never; 5 = always), with higher scores indicating greater clinical severity. Scores exceeding the normal range, as described in Table 1, are considered indicative of a sleep disorder.
- Emotional Disorders:
  The Educational-Clinical Questionnaire: Anxiety and Depression (CECAD) is a validated instrument designed to assess emotional disorders in children and adolescents aged 7 to 18 years. Due to its robust psychometric properties and widespread use in early screening, it is commonly applied to identify symptoms of anxiety and depression in pediatric populations. It consists of 50 questions that assess the presence of depression (29 questions), anxiety (20 questions), worthlessness (8 questions), irritability (6 questions), cognitive disturbances (7 questions), and psychophysiological symptoms (16 questions) and 1 question assessing clinical aspects, scored using a 5-point Likert scale (never, almost never, sometimes, almost always, always) [23]. The CECAD has demonstrated good reliability and validity and enables early detection of individuals at psychological risk, facilitating timely interventions [24]. The survey responses were processed through the virtual analysis platform recommended by the authors [24], ensuring standardized interpretation of results. Scores above the normative mean were classified as a risk factors for emotional disorders (Figure 2).

In both questionnaires, the timeframe for completion depended mostly on the participant and whether they had the option to ask questions about any issues that arose.

1. History Taking/Anamneses:
2. Each patient underwent a structured clinical interview conducted by a trained researcher in a dental office setting. The interview lasted approximately 35 min and included questions regarding the following:
  - Jaw pain:
    Discomfort located in the temporal region, preauricular area, or around the ear, as well as the presence of headaches. Patients were asked about the onset, duration, and activities that aggravated or relieved the pain.
  - Joint noises:
    The presence of clicking, popping, or crepitus during mandibular movements, including questions about their frequency, duration, and specific jaw movements that provoked them.
  - Jaw locking or limited movement:
    Episodes of mandibular blockage during opening or closing, including duration and how the patient resolved the restriction.
  - Clinical examination:
    A calibrated examiner assessed the clinical presence or absence of temporomandibular dysfunction signs. This included joint sounds detected via auscultation or palpation during lateral and protrusive movements, muscle tenderness upon firm bilateral digital palpation of the masseter and temporalis muscles, mandibular deviations during opening and closing (corrected or uncorrected), and restricted jaw opening. Pain intensity upon palpation was recorded using a six-point visual analog scale, ranging from 0 (no pain) to 5 (very intense pain), with 3 indicating a moderate pain response.
    In accordance with the DC/TMD diagnostic criteria, a definitive diagnosis of temporomandibular disorders was established only when the discomfort reported during the anamnesis could be reproduced during clinical examination. Muscle or joint pain was considered positive for TMDs if the same area elicited similar discomfort upon palpation or functional testing, thus ensuring consistency between subjective reports and objective findings.
  - Clinical examination system:
    The exam was performed by a different investigator to the researcher recording patient history. Each patient was seated in a dental chair (Sinius Sirona^®^) at a 45° angle and the clinical examination was divided into two parts: extraoral and intraoral.
    This examination determined the clinical presence or absence of joint sounds, pain, mandibular deviation, or limited jaw opening. Joint sounds were diagnosed by audible sound or palpation of the TMJ, while joint pain was identified through child-reported discomfort during joint palpation. Muscle pain was assessed by applying firm bilateral digital pressure over the largest portion of each masticatory muscle. Mandibular deviation was noted when the jaw deviated from the midline during opening and closing. A maximum interincisal opening of less than 35 mm was considered limited opening. Each alteration was recorded as present or absent on the standardized study form, which was based on the official Spanish-language version of the DC/TMD assessment instruments, translated and validated by the International Network for Orofacial Pain and Related Disorders Methodology (INfORM) [9]. To avoid confounding effects from dental intervention, all clinical evaluations were performed at least four weeks after dental treatment, ensuring that findings reflected true temporomandibular dysfunction rather than transient postoperative effects.

#### 2.2.4. Reliability and Reproducibility

Data collection was conducted by two experienced researchers. To ensure diagnostic consistency and minimize inter-observer variability, the two clinicians responsible for performing the examinations underwent a structured calibration process prior to initiating data collection. This process consisted of two phases. First, both examiners participated in a theoretical–practical training session based on the official Diagnostic Criteria for Temporomandibular Disorders (DC/TMD) protocol, during which the key diagnostic steps were standardized, and consensus was reached on interpretation criteria for muscle tenderness, joint sounds, and mandibular movement patterns.

Subsequently, in the second phase, both researchers independently examined a pilot group of 10 pediatric patients not included in the final study sample. Their results were compared using Cohen’s kappa coefficient to determine the level of inter-examiner agreement for each variable assessed. A kappa value of 0.85 or higher was considered acceptable for proceeding with data collection. The calibration process was supervised by two senior clinicians with expertise in orofacial pain and TMD diagnosis. Additionally, periodic meetings were held throughout the study to re-evaluate procedures and resolve any discrepancies in the interpretation of clinical signs.

Twenty percent of the patients who participated in the study were randomly recalled to repeat the tests.

#### 2.2.5. Statistical Analysis

The analysis was carried out using IBM SPSS^®^ version 26. Frequencies and percentages were applied to summarize qualitative variables, while measures of central tendency were used for quantitative variables. The prevalence ratio was calculated as a measure of association, and its statistical significance was assessed using the chi-square test. The significance level was set at *p* < 0.05. A 95% confidence interval was computed for the analysis. The effect size was calculated using Cramér’s V, where 0 indicates no association between emotional and sleep disorders and the signs and symptoms of TMDs, whereas a value of 1 indicates a maximum association between these variables [25].

## 3. Results

### 3.1. Population Description

The population comprised children with a mean age of 10.89 ± 2.19 years who attended different dental clinics in a population belonging to the province of Guadalajara in the Community of Castilla-La Mancha during the period 2023–2024.

TMD prevalence was 54%. Among these cases, 26% presented muscle pain, 14% joint pain, and 14% both types of pain. Of the patients diagnosed with TMDs, 75% exhibited joint clicks, 11.1% experienced joint locking, and 81% showed corrected deviation during mouth opening and closing.

### 3.2. Relationship Between Sleep Disorders and Temporomandibular Disorders

The data from the Bruni et al. questionnaire showed that difficulties in initiating and maintaining sleep were significantly related to both muscle and joint pain. Specifically, patients with muscle pain had a mean score of 26.53 (SD = 30.06), while those with joint pain had a mean score of 12.14 (SD = 2.79), with a significant *p*-value of 0.017 and a 95% confidence interval, indicating that children with sleep initiation and maintenance problems are significantly more likely to report painful TMD symptoms. These comparative values between groups are reflected in Table 2, which details the distribution of sleep disturbance scores by clinical manifestation.

In addition, corrected mandibular deviation during mouth opening was also influenced by sleep onset and maintenance disturbances, with a mean score of 18.71 (SD = 21.06) and a *p*-value of 0.047 with a 95% confidence interval, further supporting the link between sleep quality and functional mandibular alterations. This comparative analysis including symptomatic and asymptomatic participants is detailed in Table 3.

In contrast, other sleep disorders such as sleep breathing disorders, arousal disorders, and hyperhidrosis do not show statistically significant associations with the presence of temporomandibular disorders, as evidenced by *p*-values above 0.05 in most comparisons, as shown in Table 2 and Table 3.

### 3.3. Impact of Emotional Disorders on TMJ

The analysis of the relationship between the CECAD (Depressive Affect Quality Assessment Questionnaire) and the presence of temporomandibular disorders indicates that anxiety plays an important role. Patients with muscle pain had a mean anxiety score of 44.87 (SD = 11.85), whereas those without symptoms had a mean of 36 (SD = 10.78), with a significant *p*-value of 0.03. Furthermore, physiological changes resulting from stress showed a statistically significant relationship with the presence of muscle and joint pain, with *p*-values of 0.008 and 0.007, respectively. This reinforces the hypothesis that psychological stress can exacerbate TMJ symptoms.

On the other hand, other psychological factors such as negative thinking, worthlessness, and irritability did not show a statistically significant relationship with the presence of clicking, joint locking, or corrected deviations (Table 4 and Table 5).

### 3.4. Associations Between the BRUNI Sleep Quality Index and TMD

The results obtained from the BRUNI index reveal that 78.13% of patients with TMDs experience difficulty in initiating and maintaining sleep, with a prevalence ratio (PR) of 3.57 and a significant *p*-value of 0.041. It was also observed that 65.63% of patients had a high score on the BRUNI index, suggesting that poor sleep quality could be associated with TMJ dysfunction (Table 6).

### 3.5. Association Between Emotional Disorders and TMDs

The data in Table 7 on the association of the CECAD with the TMJ also reveal that patients with high scores for emotional stress tend to report more TMJ symptoms, with a statistically significant difference compared to those with less emotional distress. *p*-values less than 0.05 indicate that the impact of stress not only affects pain perception but also mandibular function in general.

On the other hand, other psychological factors such as negative thinking, worthlessness, and irritability do not show a statistically significant relationship with the presence of clicking, joint locking, or corrected deviations.

## 4. Discussion

TMDs represent a term that encompasses a myriad of signs and symptoms affecting the masticatory muscles, the temporomandibular joint, or both [26] and are often accompanied by psychological factors such as stress, anxiety, and irritable bowel syndrome (IBS) [27]. Therefore, we have identified the prevalence of these disorders and their association with sleep disturbances, stress, and anxiety in children.

The results obtained in this study reveal a high prevalence of clinical manifestations of temporomandibular disorders (TMDs) in children and adolescents, reaching 54%, a value notably higher than that reported by Rentsch et al. (18.8%), Al-Khotani et al. (27.2%), Mara de Paiva et al. (34.9%), Ebrahimi et al. (34.7%), and Restrepo et al. (40%) [2,19,28,29,30]. Díaz et al. reported a 43.4% prevalence of TMJ problems, citing that 72.3% of the cases presented a single disorder, 20.4% two TMDs, and 7.3% more than two TMDs, in similar populations. This coincides with the values reported by Eraslan et al. (66.8%) in a young adult population [31,32], and those reported by Jie Lei et al. (61.4%) in Asian adolescents [33]. While the study by Taneja et al. in an Indian adolescent population used both the FAI and the CD/TMD questionnaire, it reported a 51% prevalence of signs and symptoms [34]. The discrepancies observed between these studies and the present one may be due to key methodological differences, such as the diagnostic criteria used, the size and demographic characteristics of the populations studied, and the environment in which the studies were conducted. For example, the study by Al-Khotani et al. and the study by Eraslan used less strict questionnaires (self-test) for the identification of TMDs [19,32]; this could explain the lower percentage of prevalence reported in those studies.

Supporting this trend at the population level, a comprehensive global meta-analysis estimated the overall prevalence of TMDs at 34%, with significant geographic variability: 47% in South America, 33% in Asia, and 29% in Europe. In children and adolescents, the worldwide prevalence is approximately 27%, with notable differences between regions—North America showing the highest (37%) and Europe the lowest (18%) [17]. These disparities underscore the importance of developing geographically contextualized screening strategies and highlight how regional, cultural, and demographic factors may influence the identification and reporting of TMDs.

In contrast, the percentage of patients who presented joint sounds in our study (75%) is more consistent with the findings of Pimenta et al. (68% in women and 67% in men) [35]. This fact suggests that, regardless of the differences in the methodologies used, certain clinical signs, such as joint sounds, seem to remain a frequent and relatively constant marker among different studies.

The study by Aravena et al. reported that four out of ten adolescents reported some type of temporomandibular pain or discomfort, the majority of whom were female, a finding that agrees with our results regarding the higher prevalence of TMDs in females. Furthermore, Aravena et al. used the CD/TMD questionnaire for the assessment of TMDs, finding a prevalence of 26.88% and a male/female ratio of 1:1.27 (*p* = 0.24) [7]. In comparison, our study reported a slightly higher proportion, which could be due to cultural factors or differences in pain perception.

A meta-regression study analyzing global data—including European cohorts—reported a 13.7% pooled co-occurrence of bruxism and TMDs in Europe and found that a higher proportion of female participants was significantly associated with increased co-occurrence rates [36]. These results support our findings on gender differences and the influence of psychosocial factors in TMDs.

It is obvious that the instruments used for the detection of TMDs influence the prevalence results; therefore, when discussing data from different studies, this aspect must be taken into account. In this regard, the study by Mendiburu-Zavala found that 74.3% of adolescents presented some degree of TMDs [37]. On the other hand, a study conducted by Torul et al. in a Turkish pediatric population found that 19.7% of participants presented symptoms of TMDs according to the Fonseca Anamnestic Index (FAI), while 38.8% showed at least one symptom on clinical examination [38].

Some researchers note that parafunctional habits significantly increase the risk of TMDs (OR = 4.24; 95% CI 1.64–10.93), as do anxiety symptoms (OR = 1.09; 95% CI 1.02–1.16) [38]. These results are consistent with our findings on the impact of parafunctional habits and psychological factors on the development of TMDs in pediatric populations.

A study conducted by Mendiburu-Zabala et al., which compared two Mexican populations of young adults, found a moderately significant correlation (0.316) between poor quality of sleep and perceived stress [37].

In addition, in our study, a significant proportion of participants reported orofacial pain and headaches associated with TMDs, which is consistent with the study by Restrepo et al., in which 25.5% of the study population presented pain, with muscle pain being the most common symptom [30]. In the same line, in the study conducted by Rentsch, one of the most common symptoms was muscle pain (6.8%) [2]. The higher prevalence of pain in women was also a finding shared by both studies, which underlines the importance of considering gender as a risk factor.

The pain reported by our participants was primarily associated with psychological factors, such as anxiety and stress. Studies such as that by Marpaug et al. also highlight that emotional factors double the risk of developing TMDs [12], reinforcing the need for a multidisciplinary approach to treatment.

Our findings also indicate a high frequency of disc displacement and joint pain, results comparable with those of a study comparing adolescent populations in China and Germany. In that study, the overall prevalence of TMDs was 13.9%, with no significant differences between the two populations, although pain was higher in Chinese adolescents (OR 1.6–6.5) [39]. The similarity in reported symptoms reinforces the need to implement standardized diagnostic strategies that allow for better comparison across cultural settings.

However, in cases where patients were unaware of the pain, an association was observed between this pain and parafunctional habits, such as bruxism; this association was also significant in our study. TMDs and bruxism have been linked as they both share a multifactorial etiology [18,36]. Although we did not have a dedicated category for bruxism, we took into consideration many of the classic symptoms associated with it such as muscle pain and complaints of a sore jaw or face and psychological factors such as depression and anxiety that are linked to a higher likelihood of bruxism. In the scientific literature, Torul et al. found a prevalence of bruxism of 84.3% in children with TMDs; Taneja et al. found statistically significant differences in the association between bruxism and TMDs, with an OR of 5.87 [34,38]. Subjects with bruxism showed more clinical signs of TMDs than those without, highlighting the need for early interventions to correct these habits and prevent long-term complications.

A recent systematic review and meta-analysis further confirmed that individuals with bruxism have significantly increased odds of developing TMDs. Specifically, awake bruxism was associated with a 2.51-fold increase in TMD risk and sleep bruxism with a 2.06-fold increase [18]. These findings reinforce the role of bruxism as a major comorbid factor in the development of TMDs.

Building on this, bruxism is widely considered one of the most important contributing factors to functional disorders of the temporomandibular joint, and it is strongly associated with underlying psychological and emotional disturbances.

In this regard, the wide confidence intervals in our results may be due to the functional alteration of mastication that these patients present and to other altered functions such as oral breathing. In this study, pain detected during clinical examination was significantly associated with the presence of parafunctional habits such as bruxism.

During clinical examination, 81.5% of patients with symptoms presented a corrected deviation opening pattern, while 11.1% depicted a history of joint block. The high prevalence of mandibular deviation found is consistent with the results of other studies, such as those of Sena et al., who reported that disc displacement is one of the most common clinical manifestations in children and adolescents [1]. In previous studies, disc displacement with reduction has been observed to be the most frequent type of joint dysfunction in children and adolescents. The study by Rentsch et al. found a prevalence of 10.2% of disk displacement with reduction, DDwR, which is consistent with our findings [2]. Furthermore, the high frequency of mandibular deviation during opening suggests the need for more detailed clinical evaluations to detect early functional alterations and prevent long-term complications.

The results show that patients with difficulty initiating or maintaining sleep are 3.6 times more likely to develop TMDs. This finding is consistent with the study by Jie Lei et al., which found a 27% comorbidity between sleep disorders and TMDs [33]. Furthermore, studies such as that of Yazıcıoğlu et al. highlight that factors such as mouth breathing and sleeping with the mouth open are independent predictors of TMDs, while sleeping with the mouth closed acts as a protective factor [40].

Furthermore, the relationship between sleep quality and TMDs is also observed in the high prevalence of nocturnal bruxism in participants with TMDs. Previous studies have suggested that nocturnal bruxism acts as a significant risk factor, increasing the risk of joint and muscle damage [41].

Sleep disorders and psychological stress are correlated with TMDs [33]. In our study, participants with high scores on the CECAD questionnaire, indicative of emotional disorders, presented clinical manifestations of TMDs up to 5.6 times more than those without these disorders. This result is in line with the study by Marpaug et al., which showed that stress and sadness double the risk of developing TMDs [12]. Similarly, Restrepo et al. found that the relationship between TMDs and anxiety and depression was statistically significant (*p* = 0.04 for anxiety and *p* = 0.03 for depression); notably, 43.3% of adolescents reported anxiety problems [30]. Somatization was higher in males, although there were no statistically significant differences between genders when compared with depression. Moreover, according to the results reported by Jie Lei et al., one in three subjects with some symptoms of TMDs experienced sleep disorders, depression, and stress [33]. The Mendiburu-Zavala study (where the population is from) found that 51.9% of patients with TMDs presented some degree of depression [37]. They also highlighted that severe depression was more prevalent in early adolescence, a result that supports our observation of the strong association between emotional factors and TMDs. In the study by Jie Lei et al. in Asian adolescents, 65.2% experienced anxiety [33]. Furthermore, the study by Torul et al. highlighted that anxiety increases the risk of TMDs, with an OR of 1.09 (95% CI 1.02–1.16) [38]. The high comorbidity between anxiety and TMDs underscores the importance of including emotional management as an integral part of treatment.

In prevalence studies of TMDs, it is worth noting that they have been accompanied by a high prevalence of parafunctional habits. The study by Rentsch et al. in Swiss children aged 7 to 14 years showed a high prevalence of onychophagia (37.7%), teeth clenching (32.2%), and bruxism/grinding (25.5%) [2].

The study by Jin et al. on Chinese adolescents with TMDs who received physical therapy shows complementary findings. Around 60% of the 286 patients treated reported short-term symptom improvement after personalized physical therapy, with significant improvements in pain intensity, maximum mouth opening, and decreased joint sounds. In the long term (30 months on average), 94% of patients had symptom improvement, although 52.8% experienced persistent mild symptoms, such as joint noises (40.9%) and occasional pain (26.9%). Parafunctional habits and an insufficient number of therapy sessions were significantly associated with persisting symptoms (OR = 4.118 and OR = 0.899, respectively). This study reinforces the need to correct harmful habits and ensure an adequate number of treatment sessions to improve long-term prognosis [42].

Among the limitations of this study are the sample size and the lack of a detailed division of participants by age group and socioeconomic status, which could have allowed for a more detailed analysis of the influence of these variables on the development of TMDs. There is also a self-selection bias due to the fact that a community primary care center was selected, which may have influenced more participants with TMDs to decide to participate in the study. All the participants were referred from either private or public dental clinics to participate in the study as long as they conformed to the age range requirement. However, there is always some possibility of participation bias as most of the participants who agreed on participating in the study could have been motivated to participate if they suffered from TMJ pain prior to signing up to the study. This bias could hinder the ability to extrapolate the results to the general population.

In addition, in order to minimize the effect of possible confounding factors, a balanced number of boys and girls was sought. In addition, the sample represents a geographical area characterized by a relatively homogeneous socioeconomic level, helping reduce the variability attributable to this factor.

This study has several strengths, such as the inclusion of a wide range of associated factors, including bruxism, stress, and anxiety. Furthermore, the use of standardized diagnostic tools allowed for an accurate assessment of the clinical manifestations of TMDs, which contributes to the validity of the results.

It is essential to conduct longitudinal studies that allow for the establishment of causal relationships between emotional factors, sleep disorders, and TMDs in pediatric populations. Likewise, it is recommended that researchers develop standardized methodologies that include uniform diagnostic criteria and the integration of more specific tools to assess risk factors associated with TMDs. Future research should also focus on therapeutic interventions aimed at improving sleep quality and reducing emotional stress, with the goal of preventing and managing TMDs in children and adolescents.

Furthermore, it is necessary to further study parafunctional habits, such as bruxism, and their relationship with TMDs, as well as evaluate the impact of educational and preventive programs that promote oral and emotional health at these early ages. These lines of research will contribute to the development of more effective strategies for the prevention and treatment of TMDs, thereby improving the quality of life of affected children and adolescents.

## 5. Conclusions

The present study revealed that temporomandibular disorders (TMDs) are common among children and adolescents, with 54% of participants presenting at least one clinical sign or symptom. Emotional disturbances and sleep problems were also prevalent, affecting 41% and 56% of participants, respectively.

The strongest associations with TMD symptoms were observed in children experiencing physiological stress responses and those with difficulties initiating and maintaining sleep. Children reporting muscle pain showed significantly higher levels of sleep-related problems compared to asymptomatic individuals. In contrast, other conditions such as breathing disorders and excessive sweating were not associated with TMDs. Overall, the analysis revealed that children with emotional disorders were over five times more likely to exhibit TMD symptoms, while those with sleep disturbances had a more than threefold increased likelihood.

These findings underscore the need for a holistic diagnostic approach to TMDs that incorporates both emotional wellbeing and sleep quality. It is recommended that pediatric dentists integrate validated psychological and sleep assessment tools into routine clinical practice, particularly when managing patients with pain or functional jaw limitations. Interdisciplinary collaboration among dentists, psychologists, and sleep specialists may significantly enhance early detection and improve treatment outcomes in pediatric TMD cases.

## Figures and Tables

**Table 1:** Normal sleep disorder scores.

Disorders	Normal Range
Initiating and Maintaining Sleep	9.9
Sleep Breathing Disorders	3.8
Arousal/Nightmare	3.3
Sleep/Wake transitions	8.1
Excessive Somnolence	7.1
Sleep Hyperhidrosis	2.9
Global Score	39

**Table 2:** Sleep disturbance scores and presence of symptoms.

Factors	Absence of Manifestation Mean ± SD	Pain Muscle Mean ± SD	Pain Joint Mean ± SD	Pain Both Mean ± SD	*p*-Value
Initiating and Maintaining Sleep	11.33 ± 3.53	26.53 ± 30.06	12.14 ± 2.79	26.00 ± 33.69	***0.017***
Sleep Breathing Disorders	4.22 ± 2.10	4.33 ± 2.47	3.57 ± 0.79	6.14 ± 4.06	*0.449*
Arousal Disorders	3.56 ± 0.70	4.93 ± 2.25	4.14 ± 1.46	4.29 ± 2.36	*0.053*
Sleep/Wake Transition Disorders	9.17 ± 2.41	12.07 ± 6.31	8.71 ± 2.87	12.86 ± 7.01	*0.375*
Excessive Somnolence	7.94 ± 3.83	10.13 ± 5.05	8.57 ± 2.94	9.14 ± 3.53	*0.154*
Hyperhidrosis	2.78 ± 1.11	4.53 ± 3.50	2.29 ± 0.76	3.71 ± 2.93	*0.336*
Sleep Disturbance	39.00 ± 10.10	57.73 ± 23.75	39.43 ± 8.50	56.14 ± 26.39	***0.020***

**Table 3:** Sleep disturbance scores and presence of signs.

Factors	Absence of Manifestation Mean ± SD	Click Mean ± SD	*p*-Value	Joint Blockage Mean ± SD	*p*-Value	Corrected Deviation Mean ± SD	*p*-Value
Initiating and Maintaining Sleep	11.33 ± 3.53	19.79 ± 22.97	*0.119*	33.20 ± 37.01	0.158	18.71 ± 21.06	***0.047***
Sleep Breathing Disorders	4.22 ± 2.10	4.43 ± 2.74	*0.318*	4.00 ± 1.73	*0.738*	4.41 ± 2.58	*0.504*
Arousal Disorders	3.56 ± 0.70	4.11 ± 1.91	*0.482*	5.20 ± 3.35	*0.476*	4.00 ± 1.76	*0.223*
Sleep/Wake Transition Disorders	9.17 ± 2.41	10.61 ± 5.12	*0.836*	14.40 ± 3.58	***0.016***	10.85 ± 4.59	*0.086*
Excessive Somnolence	7.94 ± 3.83	8.93 ± 4.45	*0.890*	12.60 ± 3.29	***0.012***	9.47 ± 4.53	*0.138*
Hyperhidrosis	2.78 ± 1.11	3.36 ± 2.56	*0.637*	3.60 ± 3.58	*0.564*	3.29 ± 2.34	*0.905*
Sleep Disturbance	39.00 ± 10.10	48.64 ± 21.27	*0.618*	64.80 ± 25.19	***0.017***	48.29 ± 19.40	*0.102*

**Table 4:** CECAD scores and presence of symptoms.

CECAD		Absence of Manifestation Mean ± SD	Click Mean ± SD	Joint Blockage Mean ± SD	Corrected Deviation Mean ± SD	*p* ^1^
Depression	Ptj ^2^ Pm ^3^	52.78 ± 16.01 44.94 ± 9.13	66.88 ± 19.75 51.47 ± 10.40	55.14 ± 15.64 46.14 ± 9.58	58.43 ± 12.18 49.71 ± 6.02	*0.055* *0.091*
Anxiety	Ptj ^2^ Pm ^3^	36.00 ± 10.78 45.33 ± 8.58	44.87 ± 11.85 53.73 ± 9.00	34.43 ± 12.46 44.00 ± 9.66	44.57 ± 12.54 52.14 ± 10.85	***0.030*** ***0.016***
Worthlessness	Ptj ^2^ Pm ^3^	14.50 ± 3.97 46.78 ± 7.70	19.00 ± 6.40 52.67 ± 9.69	14.86 ± 4.71 46.86 ± 9.92	14.71 ± 4.23 47.71 ± 6.13	*0.093* *0.098*
Irritability	Ptj ^2^ Pm ^3^	11.78 ± 5.25 47.28 ± 9.94	14.73 ± 5.71 52.53 ± 10.70	12.57 ± 3.60 49.57 ± 7.09	16.14 ± 4.49 56.86 ± 7.22	***0.043*** ***0.032***
Negative Thinking	Ptj ^2^ Pm ^3^	13.61± 5.41 46.22 ± 9.49	68.87 ± 5.95 51.87 ± 10.32	16.71 ± 11.32 45.86 ± 7.86	14.14 ± 4.30 48.00 ± 7.30	*0.203* *0.183*
Physiological Changes	Ptj ^2^ Pm ^3^	29.17 ± 7.96 44.89 ± 8.13	37.73 ± 9.19 54.07 ± 8.71	28.43 9.90 43.57 9.52	35.29 ± 8.65 51.14 ± 8.99	***0.008*** ***0.007***

^1^ *p*-value for clicks, joint blockage, and correlation deviation. ^2^ Established score in this investigation. ^3^ Normal value.

**Table 5:** CECAD scores and presence of signs.

CECAD		Absence of Manifestation Mean ± SD	Pain Muscle Mean ± SD	*p* ^1^	Pain Joint Mean ± SD	*p* ^1^	Pain Both Mean ± SD	*p* ^1^
Depression	Ptj ^2^ Pm ^3^	52.78 ± 16.01 44.94 ± 9.13	57.75 ± 20.25 46.46 ± 11.25	*0.907* *0.740*	69.40 ± 26.39 51.60 ± 13.52	*0.201* *0.308*	55.03 ± 17.57 45.85 ± 10.25	*0.248* *0.275*
Anxiety	Ptj ^2^ Pm ^3^	40.14 ± 13.01 49.07 ± 10.71	44.87 ± 11.85 53.73 ± 9.00	*0.531* *0.564*	44.00 ± 15.60 51.00 ± 12.19	*0.391* *0.549*	38.00 ± 11.37 47.35 ± 9.49	*0.454* *0.441*
Worthlessness	Ptj ^2^ Pm ^3^	16.00 ± 6.54 47.36 ±11.10	19.00 ± 6.40 52.67 ± 9.69	*0.906* *0.829*	20.00 ± 8.97 52.80 ± 13.03	*0.256* *0.331*	15.09 ± 5.91 46.62 ± 10.10	*0.136* *0.156*
Irritability	Ptj ^2^ Pm ^3^	12.93 ± 4.68 49.29 ± 8.86	14.73 ± 5.71 52.53 ± 10.70	*0.969* *0.953*	15.40 ± 6.07 54.40 ± 10.50	*0.299* *0.315*	12.59 ± 4.57 49.18 ± 8.84	*0.326* *0.416*
Negative Thinking	Ptj ^2^ Pm ^3^	13.79 ± 5.83 46.25 ± 10.83	68.87 ± 5.95 51.87 ± 10.32	*0.255* *0.378*	16.60 ± 8.26 52.00 ± 12.47	*0.661* *0.582*	13.50 ± 5.33 46.06 ± 9.90	*0.073* *0.128*
Physiological Changes	Ptj ^2^ Pm ^3^	33.50 ± 10.05 49.21 ± 10.05	37.73 ± 9.19 54.07 ± 8.71	*0.318* *0.337*	35.00 ± 12.79 49.60 ± 11.80	*0.650* *0.709*	31.44 ± 8.72 47.29 ± 9.20	*0.441* *0.472*

^1^ *p*-value for pain scores. ^2^ Established score in this investigation. ^3^ Normal value.

**Table 6:** Association between sleep disorders and TMDs.

Sleep Disorders	% *	PR	*p*-Value	95% CI Lower	95% CI Upper	Cramer’s V
Initiating and maintaining sleep	78.13	3.570	***0.041***	1.026	12.43	0.289
Sleep breathing disorders	25.00	1.167	*0.825*	0.290	4.590	0.031
Arousal disorders	62.50	2.083	*0.217*	0.640	6.730	0.175
Sleep/wake transition disorders	40.63	0.855	*0.793*	0.270	2.750	0.037
Excessive somnolence	37.50	2.100	*0.266*	0.560	7.880	0.157
Hyperhidrosis	34.38	1.830	*0.368*	0.490	6.930	0.127
Sleep disorders	65.63	2.390	*0.145*	0.730	7.780	0.206

* The percentage of participants that presented x sleep alteration and DTM.

**Table 7:** Association between emotional disorders and TMDs.

CECAD	%	PR	*p*-Value	95% CI Lower	95% CI Upper	Cramer’s V
Anxiety	50.00	2.600	*0.126*	0.750	9.008	0.216
Depression	46.87	1.765	*0.320*	0.531	5.865	0.13
Worthlessness	46.87	1.765	*0.352*	0.531	5.865	0.132
Irritability	59.73	2,297	*0.164*	0.710	7.490	0.197
Negative thinking	37.50	1.560	*0.483*	0.450	5.470	0.099
Physiological changes	53.13	5.667	***0.012***	1.370	23.46	0.357
TOTAL	47.87	3.088	*0.085*	0.833	11.44	0.244

## Data Availability

The original contributions presented in this study are included in the article. Further inquiries can be directed to the corresponding author.
